# Systematic review of technical factors associated with persistent fistula after EUS-directed transgastric ERCP in patients with Roux-en-Y gastric bypass

**DOI:** 10.1016/j.gie.2025.03.1331

**Published:** 2025-04-08

**Authors:** Scott Esckilsen, Marla Sagatelian, Paul J. Nietert, Muhammad Usman Baig, Reem Z. Sharaiha, B. Joseph Elmunzer

**Affiliations:** (1) Division of Gastroenterology & Hepatology, Medical University of South Carolina, Charleston, South Carolina, USA; (2) College of Medicine, Medical University of South Carolina, Charleston, South Carolina, USA; (3) Department of Public Health Sciences, Medical University of South Carolina, Charleston, South Carolina, USA; (4) Division of Gastroenterology & Hepatology, Weill Cornell Medicine, New York, New York, USA

## Abstract

**Background and Aims:**

An important unintended consequence of EUS-directed transgastric ERCP (EDGE) in patients with Roux-en-Y gastric bypass is persistent fistula (PF) formation after stent removal. To better inform procedural decision-making, we wanted to elucidate the technical factors associated with PF after EDGE.

**Methods:**

We performed a systematic literature search of EMBASE, Scopus, and PubMed databases from inception until November 2024. Two authors independently extracted data on technical factors potentially associated with PF. Discrepancies were resolved by consensus. The association between PF and selected technical characteristics was evaluated.

**Results:**

Of 398 patients who underwent objective testing after EDGE, 78 (19.6%) had PF. There did not appear to be an association between PF and location of the fistula (gastrogastric vs jejunogastric) (relative risk, 1.16; 95% CI, 0.93-1.41; *P* = .14) or whether ERCP was performed at the time of fistula creation or later (relative risk, 1.03; 95% CI, 0.66-1.70; *P* = .99). The use of a larger lumen-apposing metal stent (20 mm vs 15 mm) did appear to be associated with PF (relative risk, 1.65; 95% CI, 1.02-2.72; *P* = .022). Passive (spontaneous) fistula closure after stent removal was also associated with PF formation relative to active closure (relative risk, 7.25; 95% CI, 1.08-192.5; *P* = .020). The association between stent dwell time and PF was not statistically significant according to the Wilcoxon signed rank sum test (*P* = .13) but was significant by the weighted paired *t* test (*P* = .04).

**Conclusions:**

In this systematic review, larger stent size, passive (spontaneous) closure after stent removal, and longer stent dwell time appeared to be associated with an increased risk of PF formation after EDGE.

## Graphical Abstract

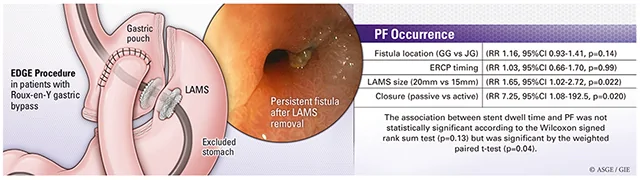

Obesity remains a major threat to public health because of its increasing prevalence and association with important medical comorbidities.^1,2^ Roux-en-Y gastric bypass (RYGB) is highly effective to treat obesity and obesity-related comorbid conditions^3^ but increases the risks of gallstone formation and related pancreaticobiliary disease, necessitating endoscopic intervention, while in parallel complicating access for ERCP.^4^ It is estimated that around 35% of patients develop gallstones during weight loss after bariatric surgery, thereby increasing their likelihood of requiring an ERCP.^5^ EUS-directed transgastric ERCP (EDGE) is an increasingly popular approach to ERCP after RYGB given its high technical success rates, shorter procedural times, and ability to perform the entire procedure in the endoscopy suite.^6-9^ The EDGE technique functionally reverses a gastric bypass by creating a fistulous tract to the excluded stomach, thereby allowing conventional peroral ERCP. After the endoscopic objective is accomplished, to reexclude the bypassed stomach, closure of the gastrogastric (GG) or jejunogastric (JG) fistula can be achieved by primary (ie, endoscopic closure) or secondary intent (ie, passive spontaneous closure).^10,11^

Although EDGE represents a major advancement in the treatment of pancreaticobiliary diseases in patients with RYGB anatomy, widespread diffusion of this procedure has been limited by the lack of robust data on long-term outcomes. One area of particular concern is the risk of persistent fistula (PF) formation after lumen-apposing metal stent (LAMS) removal, a phenomenon associated with weight regain and poor glycemic control when occurring spontaneously.^12^ The existing literature is sparse on actionable data pertaining to risk factors for PF after EDGE that might inform procedural decision-making.

We performed a systematic review to elucidate the technical factors associated with PF formation after LAMS removal in patients with RYGB undergoing EDGE. Our aim was to determine and compare the incidence of PF after EDGE according to the following technical factors: LAMS size, fistula location (GG vs JG), whether ERCP was performed during the time of initial fistula creation, LAMS dwell time, and closure approach at the time of LAMS removal.

## METHODS

### Search strategy

This systematic review was designed in accordance with standard (Preferred Reporting Items for Systematic reviews and Meta-Analyses) guidelines^13^ and was registered on PROSPERO on September 19, 2024 (registration no. CRD42 024588379). EMBASE, Scopus, and PubMed were searched to identify potentially relevant articles published from inception until November 2024. Key words used in the literature search included a combination of “EDGE,” “endoscopic ultrasound-directed transgastric ERCP,” “EDGI,” “EUS-directed transgastric intervention,” “fistula,” “persistent fistula,” and “refractory fistula.” The citation lists of potentially eligible articles were reviewed manually to identify additional publications. Additionally, the “similar articles” and “related documents” tools were used to identify other potentially eligible studies.

### Eligibility criteria

Studies were considered eligible for inclusion if they pertained to EDGE or EUS-directed transgastric intervention in patients with RYGB and provided data on the presence of PF after LAMS removal according to ≥1 of the following factors: stent size, fistula location, whether ERCP was performed during the initial session, stent dwell time, and closure technique. Individual patients were included in the analysis only if they underwent formal testing for PF after LAMS removal using EGD or imaging. In other words, patients without a follow-up assessment to evaluate for PF were not included in the numerator or denominator of any analysis. Exclusion criteria were studies on EDGE technique adapted to non–Roux-en-Y surgically altered anatomy, studies not published in English, and studies in an animal model.

### Data extraction

The following data from eligible articles were abstracted in an independent fashion by 2 authors (S.E. and M.S.): number of patients who had objective testing for presence of PF after LAMS removal, number of patients with objective fistula assessment found to have a PF, number of 15-mm versus 20-mm LAMSs used, number of GG versus JG stent locations, number of single- versus dual-session ERCPs, LAMS dwell time in patients with PF and non-PF, and closure technique used at the time of LAMS removal. Discrepancies in abstracted data were resolved by consensus, but a third investigator (B.J.E.) was available for final adjudication if necessary.

### Statistical analysis

Because there is no widely accepted definition of PF after EDGE, a PF was defined in this study as one that was identified on follow-up testing, which typically occurred weeks after LAMS removal. The associations between PF and selected technical characteristics (ie, stent size, fistula location) were assessed by pooling the study data into contingency tables, conducting Fisher exact tests, and calculating relative risks and their respective exact 95% CIs. The comparison of stent dwell times (among 6 studies that reported dwell times) was not as straightforward, because 4 studies reported medians and 2 studies reported only means. For these latter 2 studies, we chose to impute their median dwell time from their mean, and then the median stent dwell times were compared using a Wilcoxon signed rank test. Given the inconsistent nature of the way these data were reported, we conducted a sensitivity analysis using a paired *t* test of the median dwell times weighted by the study sample size. An inverse variance weighting technique, often used for meta-analyses, was not possible because the studies did not consistently report the dwell time SD and/or variance. All hypothesis tests were 2-sided, and *P* < .05 were considered statistically significant. Statistical analyses were conducted using SAS v9.4 (SAS Institute, Cary, NC, USA).

## RESULTS

### Literature search

**Figure 1.**
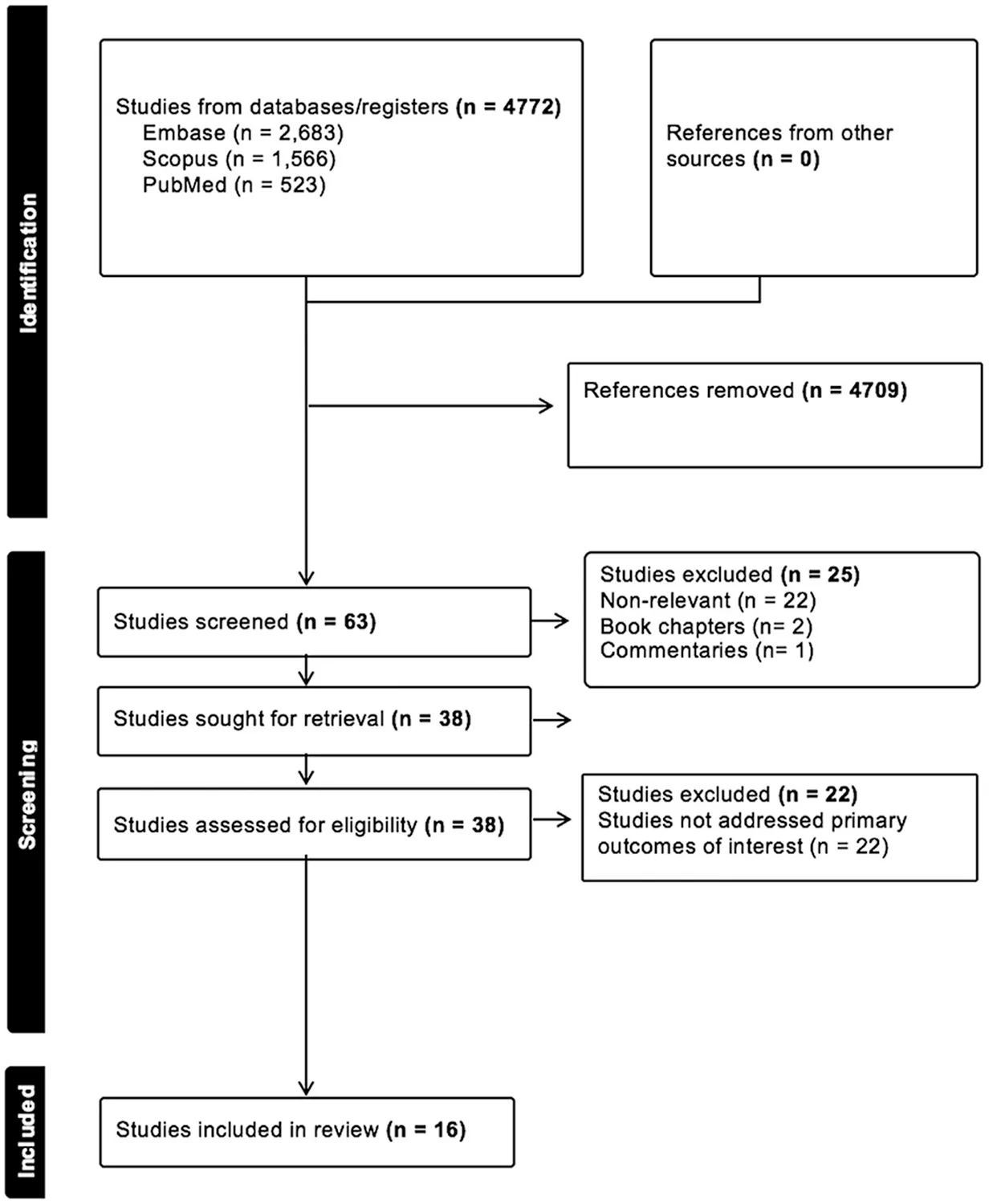
Literature search.

Searches of the EMBASE, Scopus, and PubMed databases yielded 4772 citations and 63 potentially relevant articles. Abstract and brief manuscript review of these 63 articles resulted in 38 studies appropriate for detailed review. Of these, 16 met eligibility criteria and were included in the final analysis.^6,9,11,14-26^ The remaining 22 studies were excluded because they did not address our primary outcomes of interest. There was 100% concordance between reviewers for study selection and data abstraction. A flow diagram depicting the search and selection process is provided in Figure 1.

### Characteristics of included studies

Characteristics of the 16 included studies are listed in Table 1. These studies were published between 2017 and 2024. Thirteen studies were retrospective and 3 were prospective. There were no randomized clinical trials. Seven studies were designed with the primary focus of determining factors that confer an increased risk of PF after LAMS removal.

The 16 studies included 661 patients, of whom 398 had objective testing after LAMS removal to confirm closure of the fistula with imaging or follow-up endoscopy. Of these 398 patients, 78 (19.6%) were found to have a PF.

### Quality assessment of the included studies

Quality assessment and risk of bias were analyzed using the Newcastle-Ottawa scale because all included studies were observational. It was done separately by 2 reviewers (S.E. and M.U.B.), and in the case of discrepancies a third reviewer (B.J.E.) was available for final adjudication. A score of 7 to 9 was considered low risk of bias, 3 to 6 as moderate risk of bias, and 0 to 2 as high risk of bias. The assessment was done separately for 13 cohort studies (Table 2) and 1 case-control study (Table 3). The remaining 2 studies, which were case series, were not analyzed. The overall quality of the included studies was good, and the risk of bias was low.

### Stent size

Thirteen of 16 studies were pertinent to the incidence of PF after EDGE according to stent size. Characteristics of these 13 studies are included in Table 4. All studies used either 15-mm or 20-mm LAMSs. One hundred twenty-eight patients had 15-mm LAMSs and 155 had 20-mm LAMSs. Of the 128 patients with 15-mm LAMSs, 23 developed a PF (18.0%). In comparison, of the 155 patients with 20-mm LAMSs, 46 developed a PF (29.7%). The relative risk of PF with the 20-mm stent (vs 15 mm) was 1.65 (95% CI, 1.02-2.72; *P* = .022).

### Fistula location (GG vs JG)

Six of 16 studies provided details on the incidence of PF after EDGE according to fistula location. Characteristics of these 6 studies are shown in Table 5. The gastric pouch was the most common location for LAMS deployment. One hundred thirteen patients had GG fistulas and 63 had JG fistulas. Of the 113 patients with GG fistulas, 39 developed a PF (34.5%). Comparatively, 15 of the 63 patients with JG fistulas developed a PF (23.8%). There was no statistically significant difference between PF risk and fistula location. The relative risk of GG versus JG LAMS deployment in relation to PF formation was 1.45 (95% CI, 0.88-2.65; *P* = .17).

### Single- versus dual-session ERCP

Five of 10 studies provided information on the incidence of PF after EDGE according to first- versus second-session ERCP (Table 6). Seventy-six patients had a first-session ERCP and 92 patients had a later ERCP. Of the 76 patients who had an ERCP performed at the same time as fistula creation, 24 developed a PF (31.6%). Similarly, 30 of 92 patients who had later-session ERCP developed a PF (32.6%). The relative risk of PF formation when comparing dual- versus single-session ERCP was 1.03 (95% CI, 0.66-1.70; *P* = .99).

### LAMS dwell time

Six of 16 studies provided summary statistics (eg, means, SDs, medians, IQRs) on stent dwell time, stratified by PF status. Characteristics of these 6 studies are included in Table 7. The median dwell times were higher among patients with PF, with medians ranging from 50 to 127 days across the 6 studies, when compared with patients without PF, with medians ranging from 29 to 58 days. The difference in medians was not statistically significant by the Wilcoxon rank sum test (*P* = .13) but was statistically significant by the weighted paired *t* test (*P* = .04) with an increase dwell time leading to PF.

### Closure technique at the time of LAMS removal

Six of 16 studies provided patient-specific details on the incidence of PF formation in relation to the closure approach used at the time of LAMS removal (Table 8). Forty patients did not have any form of active closure under the presumption that the fistula would spontaneously heal. Other closure techniques were double-pigtail stents placed in 11 patients, argon plasma coagulation alone in 9 patients, endoscopic suturing in 6 patients, and over-the-scope-clip placed at the time of LAMS removal in 3 patients. Of these various closure methods, 10 of 40 patients with passive spontaneous closure developed a PF (25.0%). Of the 29 patients who underwent active closure with either argon plasma coagulation, double-pigtail stent placement, endoscopic suturing, or over-the-scope clip placement, only 1 patient (who had endoscopic suturing) developed a PF (3.4%). When passive spontaneous closure was compared with any form of active closure, the relative risk of PF was 7.25 (95% CI, 1.08-192.5; *P* = .020).

## DISCUSSION

Existing literature suggests that, in expert hands, EDGE is safe and efficacious.^7,27^ However, concerns related to PF development after stent removal remain valid. The theoretical risk of weight regain because of permanent reversal of the bypass is potentially devastating to patients from both a metabolic and mental health perspective. Furthermore, the presence of a PF may increase the risk of marginal ulcer formation and chronic abdominal pain and necessitates additional procedures to reattempt closure. Thus, reducing PF remains a major priority to optimize the risk-to-benefit ratio of EDGE.

In this systematic review, we found that stent size, stent dwell time, and allowing the fistula to close by secondary intent are probably associated with PF formation, whereas the location of the fistula and the session during which ERCP is performed do not appear to be related. Because stent size, dwell time, and closure technique are modifiable, this information may inform endoscopic decision-making and are factors for which endoscopists performing EDGE should be mindful to mitigate the risk of PF and improve procedural outcomes.

In 2018, the 20-mm LAMS became commercially available and has since gained popularity for EDGE because of its lower postulated risk of stent migration during same-session ERCP,^27^ although the location of the fistula may be a more important determinant of dislodgement. Although acute stent migration events can generally be rescued endoscopically in real time, the risk of peritoneal contamination and endoscopist stress associated with urgent salvage underscore the value in avoiding such events and may justify the use of a larger LAMS if same-session ERCP is mandatory, such as in cases of ascending cholangitis or uncontrolled bile leak. However, if the indication is not urgent and the plan is to delay ERCP until after the fistula tract has matured, then it seems prudent to favor the 15-mm LAMS for the purpose of reducing PF.

Although the analysis of stent dwell time was not straightforward because of the inconsistency in how this outcome was reported, the overall findings of this systematic review suggest that longer dwell time is associated with PF, reflective of 3 of 6 included studies that observed significant differences.^11,15,20^ Therefore, until additional data are available, endoscopists should strive to minimize dwell time to the greatest extent possible while allowing sufficient time for the tract to mature before stent removal. Patients who require prolonged endotherapy, such as for pancreaticobiliary stricture remodeling, should be counseled a priori about the potentially increased risk of PF associated with a substantial dwell time.

We also found that some form of active closure appears to reduce PF relative to closure by secondary intent. Even though closure does occur spontaneously at a reasonable rate and active closure does require additional resources and time, based on available evidence, it does appear prudent to actively close EDGE fistulas at the time of stent removal to minimize the risk and consequences of PF. Unfortunately, given the very small sample size, a comparison of closure techniques (ie, suturing, clip, argon plasma coagulation, etc) was not possible. Additional research will elucidate the true value of closure and how best to achieve it.

The findings of this systematic review should be interpreted in the context of several important limitations. First, we included only patients who had follow-up testing necessary to determine whether a PF had developed. We elected to exclude patients in which serial weights were used to monitor for PF development because existing literature is inconsistent regarding the ability of weight gain to predict PF.^6,9,11,18,21,27^ Although this approach increased objectivity in the analysis, it likely exaggerated PF rates because testing was nonrandom and patients with clinical suggestion of PF may have been preferentially tested. This bias may have also led to imbalanced testing according to technical factors (eg, overtesting patients without active closure). Second, the variable reporting of studies and small sample sizes precluded adjusted analyses to evaluate the influence of other clinical and technical factors. However, qualitatively, there did not appear to be clustering of important variables. Specifically, patients who had 20-mm LAMS placement did not appear to also have longer dwell times and were not more likely to have spontaneous closure. Third, the small number of studies and sample sizes limit precision in our effect estimates, which we anticipate will be refined by additional research. Along these lines, 5 of 16 included studies contributed one-half of the total patients analyzed. In these 5 overrepresented studies, the incidence of PF was highest, potentially exaggerating the overall incidence. Also, because there was inconsistency in the nature of the summary statistics presented for stent dwell times, it was not possible to conduct an inverse variance weighted comparison of means between patients with and without PF; thus, our findings regarding higher stent dwell times among patients with PF should be considered preliminary and explored in greater detail in future studies. Finally, an inherent but major limitation of this systematic review is that the findings are based on observational studies and not randomized controlled trials.

In conclusion, this systematic review suggests that larger stent size, longer LAMS dwell time, and spontaneous closure after LAMS removal are technical factors associated with increased likelihood of PF formation after EDGE. Although the underlying mechanisms through which these technical factors are associated with PF are unknown, this information can inform procedural decision-making toward reducing the incidence of PF and its potential consequences. This systematic review also highlights the need for large-scale multicenter collaborations to answer these important questions.

## Figures and Tables

**TABLE 1.** Characteristics of included studies

Study	Study design	Total no. of patients	No. of patients tested for PF	Total patients with a PF	Overall PF rate in tested patients n/N (%)
Ngamruengphong^21^ (2017)	Retrospective cohort	13	12	1	1/12 (8.3)
Tyberg^9^ (2017)	Prospective cohort	16	8	1	1/8 (12.5)
Bukhari^6^ (2018)	Retrospective cohort	30	23	1	1/23 (4.3)
Irani^17^ (2018)	Case series	5	4	0	0/4 (0)
James^18^ (2019)	Retrospective cohort	19	11	1	1/11 (9.1)
Gutta^16^ (2019)	Retrospective cohort	19	5	0	0/5 (0)
Wang^25^ (2019)	Case series	10	7	0	0/7 (0)
Abidali^14^ (2020)	Retrospective cohort	9	9	0	0/9 (0)
Tyberg^23^ (2020)	Prospective cohort	19	13	5	5/13 (38.5)
Wang^24^ (2020)	Retrospective cohort	18	11	0	0/11 (0)
Krafft^11^ (2022)	Prospective cohort	22	22	9	9/22 (40.9)
Runge^22^ (2021)	Retrospective cohort	178	90	9	9/90 (10)
Ghandour^15^ (2023)	Case-control	75	75	25	25/75 (33.3)
Keane^19^ (2023)	Retrospective cohort	37	28	7	7/28 (25)
Kedia^20^ (2023)	Retrospective cohort	172	62	19	19/62 (30.6)
Cui^26^ (2024)	Retrospective cohort	19	18	0	0/18 (0)
Total		661	398	78	78/398 (19.6)

*PF*, Persistent fistula.

**TABLE 2.** Risk of bias summary for included cohort studies

		Selection			Comparability		Outcome		
Study	Representative exposed cohort	Selecting nonexposed cohort	Ascertainment of exposure	Outcome not at start	Comparability of cohorts	Outcome assessment	Follow-up long enough	Adequacy of follow-up	Total
Ngamruengphong^21^ (2017)	*	*	*	*		*	*	*	7/9 (low RoB)
Tyberg^9^ (2017)	*	*	*	*		*	*	*	7/9 (low RoB)
Bukhari^6^ (2018)	*	*	*	*	*	*	*	*	8/9 (low RoB)
James^18^ (2019)	*	*	*	*		*	*	*	7/9 (low RoB)
Gutta^16^ (2019)	*	*	*	*		*	*	*	7/9 (low RoB)
Abidali^14^ (2020)	*	*	*	*	*	*	*	*	8/9 (low RoB)
Tyberg^23^ (2020)	*	*	*	*		*	*	*	7/9 (low RoB)
Wang^24^ (2020)	*	*	*	*		*	*	*	7/9 (low RoB)
Krafft^11^ (2022)	*	*	*	*		*	*	*	7/9 (low RoB)
Runge^22^ (2021)	*	*	*	*		*	*	*	7/9 (low RoB)
Keane^19^ (2023)	*	*	*	*		*	*	*	7/9 (low RoB)
Kedia^20^ (2023)	*	*	*	*		*	*	*	7/9 (low RoB)
Cui^26^ (2024)	*	*	*	*		*	*	*	7/9 (low RoB)

A score of 7-9 was considered low RoB, 3-6 as moderate RoB, and 0-2 as high RoB.

*RoB*, Risk of bias.

* Requirements were met per the Newcastle-Ottawa scale.

**TABLE 3.** Risk of bias summary for included case-control studies

		Selection			Comparability		Exposure		
Study	Adequate case definition	Representativeness of cases	Selection of controls	Definition of controls	Comparability of cases and controls	Ascertainment of exposure	Same method of ascertainment for cases and controls	Nonresponse rate	Total
Ghandour^15^ (2023)	*	*	*	*	**	*	*	*	9/9 (low RoB)

A score of 7-9 was considered low RoB, 3-6 as moderate RoB, and 0-2 as high RoB.

*RoB*, Risk of bias.

* Requirements were met per the Newcastle-Ottawa scale.

**TABLE 4.** Stent size

Study	Study design	No. of patients tested for PF	LAMS size and no. used	No. of PFs found per LAMS size	Overall PF rate in 15-mm LAMSs n/N (%)	Overall PF rate in 20-mm LAMSs n/N (%)
Ngamruengphong^21^ (2017)	Retrospective cohort	12	15-mm, 12	1	1/12 (8.3)	
Tyberg^9^ (2017)	Prospective cohort	8	15-mm, 8	1	1/8 (12.5)	
Bukhari^6^ (2018)	Retrospective cohort	23	15-mm, 23	1	1/23 (4.3)	
Irani^17^ (2018)	Case series	4	15-mm, 4	0	0/4 (0)	
James^18^ (2019)	Retrospective cohort	11	15-mm, 11	1	1/11 (9.1)	
Gutta^16^ (2019)	Retrospective cohort	5	15-mm, 5	0	0/5 (0)	
Wang^25^ (2019)	Case series	7	15-mm, 7	0	0/7 (0)	
Tyberg^23^ (2020)	Prospective cohort	13	15-mm, 13	5	5/13 (38.5)	
Krafft^11^ (2022)	Prospective cohort	22	20-mm, 22	9		9/22 (40.9)
Ghandour^15^ (2023)	Case-control	75	15-mm, 30 20-mm, 45	11 14	11/30 (36.7)	14/45 (31.1)
Keane^19^ (2023)	Retrospective cohort	28	20-mm, 28	7		7/28 (25)
Kedia^20^ (2023)	Retrospective cohort	57	15-mm, 15 20-mm, 42	3 16	3/15 (20)	16/42 (38.1)
Cui^26^ (2024)	Retrospective cohort	18	20-mm, 18	0		0/18 (0)
Total		283	15-mm, 128 20-mm, 155	23 46	23/128 (18)	46/155 (29.7)

*PF*, Persistent fistula; *LAMS*, lumen-apposing metal stent.

**TABLE 5.** Fistula location

Study	Study design	No. of patients tested for PF	Lumen-apposing metal stent location	No. of PFs	Overall PF rate in GG location n/N (%)	Overall PF rate in JG location n/N (%)
Irani^17^ (2018)	Case series	4	GG, 4	0	0/4 (0)	
James^18^ (2019)	Retrospective cohort	11	GG, 5 JG, 6	1	0/5 (0)	1/6 (16.7)
Wang^25^ (2019)	Case series	7	GG, 2 JG, 5	0 0	0/2 (0)	0/5 (0)
Krafft^11^ (2022)	Prospective cohort	22	GG, 12 JG, 10	5 4	5/12 (41.7)	4/10 (40)
Ghandour^15^ (2023)	Case-control	75	GG, 58 JG, 17	21 4	21/58 (36.2)	4/17 (23.5)
Kedia^20^ (2023)	Retrospective cohort	57	GG, 32 JG, 25	13 6	13/32 (40.6)	6/25 (24)
Total		176	GG, 113 JG, 63	39 15	39/113, (34.5)	15/63 (23.8)

*PF*, Persistent fistula; *GG*, gastrogastric; *JG*, jejunogastric.

**TABLE 6.** Single- vs dual-session ERCP

Study	Study design	No. of patients tested for PF	ERCP stages	No. of PFs	Overall PF rate in single stage n/N (%)	Overall PF rate in dual stage n/N (%)
Irani^17^ (2018)	Case series	4	Single, 4	0	0/4 (0)	
James^18^ (2019)	Retrospective cohort	11	Single, 2 Dual, 9	1 0	1/2 (50)	0/9 (0)
Krafft^11^ (2022)	Prospective cohort	22	Single, 9 Dual, 13	4 5	4/9 (44.4)	5/13 (38.5)
Ghandour^15^ (2023)	Case-control	75	Single, 39 Dual, 36	10 15	10/39 (25.6)	15/36 (41.7)
Kedia^20^ (2023)	Retrospective cohort	56	Single, 22 Dual, 34	9 10	9/22 (40.9)	10/34 (29.4)
Total		168	Single, 76 Dual, 92	24 30	24/76 (31.6)	30/92 (32.6)

*PF*, Persistent fistula.

**TABLE 7.** Lumen-apposing metal stent dwell time

Study	Study design	No. of patients tested for PF	PF group dwell time (days)	Non-PF group dwell time (days)	Overall PF rate in tested patients n/N (%)
Irani^17^ (2018)	Case series	4	Not applicable	Mean: 28.5 Median: 29	0/4 (0)
James^18^ (2019)	Retrospective cohort	11	Median: 50	Median: 58 Mean: 124.8	1/11 (9.1)
Krafft^11^ (2022)	Prospective cohort	22	Median: 77 IQR: 42-124	Median: 35 IQR: 26-45	9/22 (40.9)
Runge^22^ (2021)	Retrospective cohort	90	Median: 50	Median: 34	9/90 (10)
Ghandour^15^ (2023)	Case-control	75	Mean: 127.3 SD: 163.6 Median: 127.3*	Mean: 48.4 SD: 42.7 Median: 48.4*	25/75 (33.3)
Kedia^20^ (2023)	Retrospective cohort	62	Mean: 85.5 SD: 55.4 Median: 85.5*	Mean: 50 SD: 30.6 Median: 50*	19/62 (30.6)

*PF*, Persistent fistula.

* The median was imputed by equating it to the mean value for these studies.

**TABLE 8.** Closure technique at the time of lumen-apposing metal stent removal

Study	Study design	No. of patients tested for PF	Primary fistula closure method	No. of PFs	Overall PF rate in spontaneous closure n/N (%)	Overall PF rate in suturing n/N (%)
Ngamruengphong^21^ (2017)	Retrospective cohort	12	APC, 3 Suture, 6 OTSC, 3	0 1 0		1/6 (16.6)
Irani^17^ (2018)	Case series	4	Spontaneous, 4	0	0/4 (0)	
James^18^ (2019)	Retrospective cohort	11	Spontaneous, 5 APC, 6	1 0	1/5 (20)	
Abidali^14^ (2020)	Retrospective cohort	9	Spontaneous, 9	0	0/9 (0)	
Wang^24^ (2020)	Retrospective cohort	11	Double-pigtail stent, 11	0		
Krafft^11^ (2022)	Prospective cohort	22	Spontaneous, 22	9	9/22 (40.9)	
Total		69	Spontaneous, 40 APC, 9 Suture, 6 OTSC, 3 Double-pigtail stent, 11	10 0 1 0 0	10/40 (25)	1/6 (16.6)

*PF*, Persistent fistula; *APC*, argon plasma coagulation; *OTSC*, over-the-scope clip.

## DIVERSITY, EQUITY, AND INCLUSION:

One or more of the authors of this paper self-identifies as an under-represented gender minority in science.

## Abbreviations:

EDGE: EUS-directed transgastric ERCP
GG: gastrogastric
JG: jejunogastric
LAMS: lumen-apposing metal stent
PF: persistent fistula
RYGB: Roux-en-Y gastric bypass
